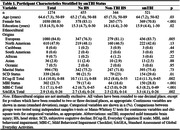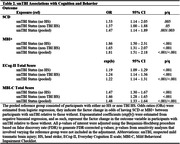# Cognitive, behavioral, and functional outcomes of suspected mild traumatic brain injury in community‐dwelling older persons without mild cognitive impairment or dementia

**DOI:** 10.1002/alz.091498

**Published:** 2025-01-09

**Authors:** Dylan X. Guan, Matthew E. Peters, G. Bruce Pike, Eric E. Smith, Zahinoor Ismail

**Affiliations:** ^1^ Hotchkiss Brain Institute, University of Calgary, Calgary, AB Canada; ^2^ Johns Hopkins University School of Medicine, Baltimore, MD USA; ^3^ Department of Clinical Neurosciences and Hotchkiss Brain Institute, University of Calgary, Calgary, AB Canada

## Abstract

**Background:**

Traumatic brain injury (TBI) is a risk factor for dementia and is linked to earlier age of onset. This study investigated whether later‐life changes in everyday cognition and behavior – risk markers of AD – could be observed in cognitively unimpaired older persons who sustained suspected mild TBI (smTBI) earlier in life and whether these cognitive and behavioral changes behavior mediated the link between smTBI and daily function.

**Method:**

Data for 1274 participants from the Canadian Platform for Research Online to Investigate Health, Quality of Life, Cognition, Behaviour, Function, and Caregiving in Aging (CAN‐PROTECT) study were analyzed. A self‐reported brain injury screening questionnaire was used to determine smTBI. Outcomes were measured using the Everyday Cognition (ECog‐II) scale, MBI Checklist (MBI‐C), and Standard Assessment of Global Everyday Activities (SAGEA). SCD was operationalized based on a score of ≥2 (i.e., consistently a little or much worse) on any ECog‐II item. MBI+ status was defined by MBI‐C total scores ≥8. Age, sex, years of education, marital status, and ethnocultural origin were balanced across exposure groups using inverse probability of treatment weighting. Weighted logistic and negative binomial regressions were used to model smTBI (exposure) associations with SCD and MBI+ statuses, and ECog‐II and MBI‐C total scores, respectively.

**Result:**

Sample characteristics are shown in Table 1. **S**ustaining smTBI throughout the life course was linked to a higher odds of participants reporting SCD or MBI, as well as higher ECog‐II or MBI‐C total scores [Table 2]. Finally, SCD partially mediated, whereas MBI+ fully mediated, the association between smTBI and SAGEA total scores; SCD and MBI+ statuses accounted for approximately 45% and 70% of the total effect, respectively [Table 3].

**Conclusion:**

Sustaining smTBI throughout the life course is linked to poorer cognition and behavior even in community‐dwelling older persons without MCI or dementia. Older persons with smTBI may benefit from early dementia risk assessment using tools that measure changes in cognition and behavior. Interventions for declining cognition and behavior may also be beneficial in this population to address functional impairment.